# TT(N)mGCCTC inhibits archaeal family B DNA polymerases

**DOI:** 10.1038/s41598-018-20127-4

**Published:** 2018-01-31

**Authors:** Shuhui Sun, Wei Guo, Jin-Shu Yang, Mengsheng Qiu, Xiao-Jing Zhu, Zhong-Min Dai

**Affiliations:** 1 College of Life Sciences, Zhejiang University, 866 Yuhangtang Road, Hangzhou, Zhejiang 310058 P.R. China; 2 Institute of Life Sciences, Key Laboratory of Organ Development and Regeneration of Zhejiang Province, College of Life Sciences, Hangzhou Normal University, Hangzhou, Zhejiang 310036 P.R. China; 3 Department of Anatomical Sciences and Neurobiology, University of Louisville, Louisville, KY40292 USA

## Abstract

The proofreading activity of the archaeal family B DNA polymerases enables PCR with high fidelity. However, thermostable proofreading DNA polymerases occasionally failed to amplify target fragment that could be amplified by Taq DNA polymerase. We have previously showed that G-rich sequences, which form G-quadruplex, can bind to and inhibit proofreading DNA polymerases. Here we showed that single-stranded oligonucleotides containing sequences of TT(N)mGCCTC can bind and inhibit archaeal family B DNA polymerases but not Taq DNA polymerase. It is very likely that TT(N)mGCCTC inhibits thermostable DNA polymerases during PCR in a single-stranded form. To the best of our knowledge, this is the first example of DNA sequence that could inhibit DNA polymerase in its single-stranded form.

## Introduction

Since the invention of polymerase chain reaction (PCR)^1,2^, great efforts have been made to improve the PCR performance by examining various thermal stable DNA polymerases (DNAPs) with different properties^3^. The DNA polymerases used for PCR are almost exclusively from family A and family B DNA polymerases^3^. The Family A DNAPs such as Taq, Tth and Tma DNAPs have 5′-3′ exonuclease activity, but normally lack 3′-5′ exonuclease activity^3–6^. Owning to the lack of 3′-5′ exonuclease activity, the family A DNAPs are inherently error prone. Contrarily, The Family B DNAPs (commonly known as high fidelity or proofreading DNAPs) such as Pfu, Kod and Tli usually have intrinsic 3′-5′ exonuclease activity, but lack 5′-3′ exonuclease activity^3,7–10^. As the 3′-5′ exonuclease activity enable the family B DNAPs to remove misincorporated nucleotides during DNA synthesis, the family B DNAPs are more accurate than the family A DNAPs.

All DNAPs share a common architectural feature consisting of thumb, palm and fingers domains^11,12^. Although the palm domain are homologous among different DNAP families, the structure of thumb and fingers domains are not homologous among different DNAP families^11^. While uracil- and inosine-containing DNA can be efficiently used by Family A DNAPs, they can cause strong inhibition to archaeal family B DNAPs^13–15^. Previously, we showed that guanine-rich sequences such as GGGGG and GGGGHGG can inhibit PCR using family B DNAPs but not Taq DNAP^16^.

Here, we found that oligonucleotides containing TT(N)mGCCTC can bind to archaeal family B DNA polymerases and inhibit their polymerase activity. Unlike the previous identified aptamers^17–19^, which only inhibit DNAPs at lower temperature, the inhibitory effect of TT(N)mGCCTC to archaeal family B DNAPs persisted during PCR, suggesting TT(N)mGCCTC inhibit archaeal family B DNAPs independent of its secondary structure.

## Results

### Inhibitory effect of a primer caused PCR failure using PrimeSTAR GXL DNA polymerase

**Figure 1:**
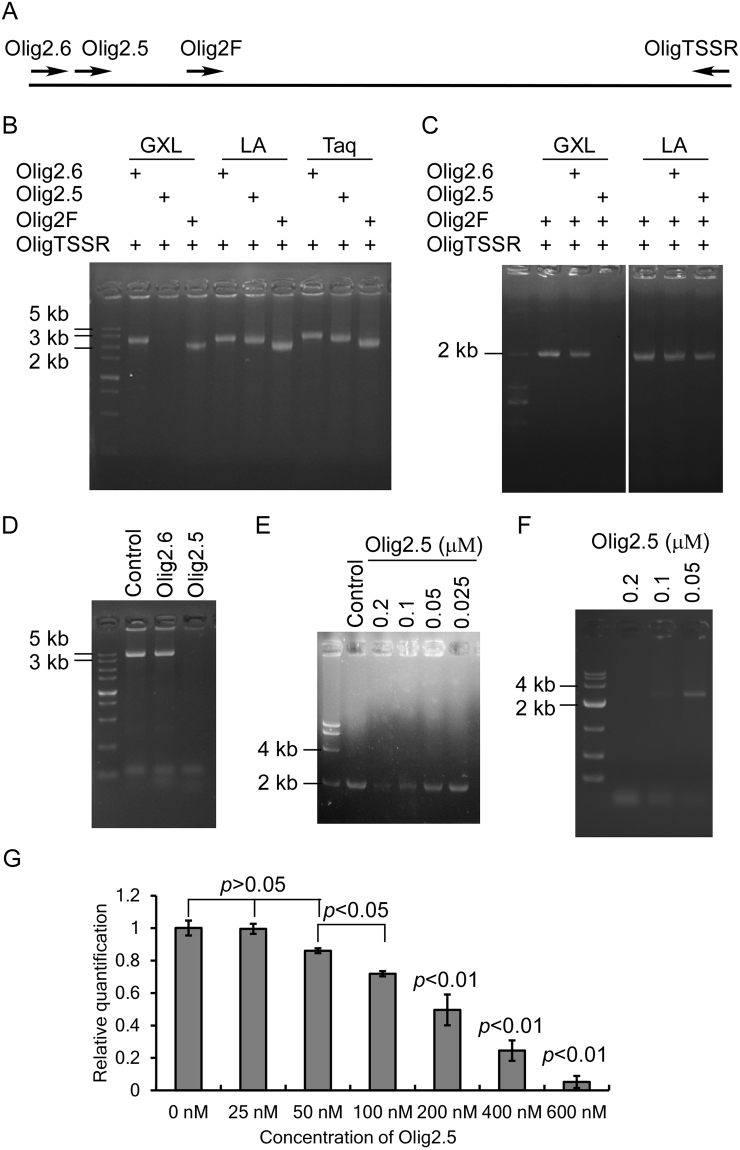
Primer Olig2.5 can inhibit PrimeSTAR GXL DNA polymerase. (**A**) Scheme of the primers used to amplify the upstream sequence of *Olig2* gene. (**B**) PrimeSTAR GXL DNAP (GXL) failed to amplify when primer Olig2.5 was used. OligTSSR was used together with Olig2.6, Olig2.5 and Olig2F to amplify the 2.6 kb, 2.5 kb and 2 kb upstream sequence of *Olig2*. LA: LA Taq DNAP. (**C**) GXL was inhibited when primer Olig2.5 but not Olig2.6 was additionally supplied. Full-length gel is presented in Supplementary Figure 1. (**D**) The amplification of a 4.5 kb fragment using GXL was also inhibited by Olig2.5. The 4.5 kb fragment was amplified by primers Ubl35armF and Ubl35armR. (**E**) Dose dependent inhibition of Olig2.5 to GXL. Various amount of Olig2.5 was additionally supplied into the PCR to amplify the 2 kb upstream sequence of *Olig2*. (**F**) Lower the concentration of Olig2.5 enabled the amplification of the 2.5 kb upstream sequence of *Olig2*. (**G**) Real-time PCR confirmed the dose dependent inhibition of Olig2.5 to GXL.

To clone the promoter region of *Olig2* from mouse genomic DNA, we failed to amplify the 2.5 kb fragment using Olig2.5 and OligTSSR as primers (all the oligonucleotides are listed in Table 1) and PrimeSTAR GXL DNA polymerase (GXL DNAP). When we used Olig2.6 and Olig2F instead of Olig2.5, we can successfully amplify the 2.6 kb and 2 kb fragments, respectively (Fig. 1B). The result suggested that the use of Olig2.5 caused the PCR failure. We then used LA Taq and Taq DNAPs instead of GXL DNAP to amplify these fragments. Both LA Taq and Taq can be used to successfully amplify the 2.6 kb, 2.5 kb and 2 kb fragments (Fig. 1B). The results showed that the PCR failure caused by Olig2.5 is specific to GXL, which also indicated that PCR failure caused by Olig2.5 is not due to inefficient primer-template annealing.

**Table 1:** Information of oligonucleotides.

Oligo name	Sequences (5′-3′)^1,2,3^	Inhibition^4^
Olig2.6	GCTCTTACGCGTGCTAGCGAAAAGTATCTCTCGGACCAAGAAG	−
Olig2	GCTCTTACGCGTGCTAGCTCCCGCATATTGTACCGCCTG	−
OligTSSR	CTTAGATCGCAGATCTCGAATAGCTGGGTGGAGGCAGC	−
Olig2qF	TCCCCAGAACCCGATGATCTT	−
Olig2qR	CGTGGACGAGGACACAGTC	−
Ubl35armF	CTCTCTTAAGGTAGCGAATTCGGTACCTAGCACACACGTCATGTGC	−
Ubl35armR	GCGATCGCCCGGATTTAAATGCTCTCCATAGTCCCACCCCTT	−
HisInTaqF	CACAGGAAACAGACCATGCACCATCATCACCACCATCACC	−
HisInTaqR	AGCATCCCGAATTCCATGCTATGGTGATGGTGGTGATGATGG	−
Olig2.5	TACCGAGCTC**TT**ACGCGTGCCTCTGACTT**GCCTC**AGAGC	+
Olig2d5	TC**TT**ACGCGTGCCTCTGACTT**GCCTC**AGAGC	+
Olig2d3	TACCGAGCTC**TT**ACGCGTGCCTCTGACTTGC	−
Olig2d51	CGCGTGCCTCTGACTT**GCCTC**AGAGC	−
Olig2d52	GCCTCTGACTT**GCCTC**AGAGC	−
Olig2d53	CTGACTT**GCCTC**AGAGC	−
Olig2d31	TC**TT**ACGCGTGCCTCTGACTT**GCCTC**AG	+
Olig2d32	TC**TT**ACGCGTGCCTCTGACTTGCCT	−
Olig2d311	TC**TT**ACGCGTGC GACTT**GCCTC**AG	+
Olig2d312	TC**TT**ACGCGT TT**GCCTC**AG	−
Olig2d313	TC**TT**ACGCG CCTCAG	−
d311R1	TC**TT**ACGCGTGC GACTT**GCCTC**A	+
d311R2	TC**TT**ACGCGTGC GACTT**GCCTC**	+
d311L1	C**TT**ACGCGTGC GACTT**GCCTC**AG	+
d311L2	**TT**ACGCGTGC GACTT**GCCTC**AG	+
d311L3	TACGCGTGC GACTT**GCCTC**AG	−
d311L4	ACGCGTGC GACTT**GCCTC**AG	−
d311LR1	**TT**ACGCGTGC GACTT**GCCTC**	+
d311LR2	**TT**ACGCGTG GACTT**GCCTC**	+
d311LR3	**TT**ACGCGT GACTT**GCCTC**	+−
d311LR4	**TT**ACGCGTGC ACTT**GCCTC**	+
d311LR5	**TT**ACGCGTGC CTT**GCCTC**	+
MutL2-1	VTACGCGT CTT**GCCTC**	−
MutL2-2	TVACGCGT CTT**GCCTC**	−
MutL2-3	**TT**BCGCGT CTT**GCCTC**	+
MutL2-4	**TT**ADGCGT CTT**GCCTC**	+−
MutL2-5	**TT**ACHCGT CTT**GCCTC**	+−
MutL2-6	**TT**ACGDGT CTT**GCCTC**	+−
MutL2-7	**TT**ACGCHT CTT**GCCTC**	+−
MutL2-8	**TT**ACGCGV CTT**GCCTC**	+−
MutL2-9	**TT**ACGCGT DTT**GCCTC**	+−
MutL2-10	**TT**ACGCGT CVT**GCCTC**	+
MutL2-11	**TT**ACGCGT CTV**GCCTC**	+−
MutL2-12	**TT**ACGCGT CTTHCCTC	−
MutL2-13	**TT**ACGCGT CTTGDCTC	−
MutL2-14	**TT**ACGCGT CTTGCDTC	−
MutL2-15	**TT**ACGCGT CTTGCCVC	−
MutL2-16	**TT**ACGCGT CTTGCCTD	−
3XGCCTC	GCCTCGCCTCGCCTC	−
TTcGCCTC	**TT**TGCGCACG CTGAA**GCCTC**	+
N8	**TT**NNNNNNNN **GCCTC**	−
N9	**TT**NNNNNNNNN **GCCTC**	+−
N10	**TT**NNNNNNNNNN **GCCTC**	+−
Bio-Olig2.5	TACCGAGCTC**TT**ACGCGTGCCTCTGACTT**GCCTC**AGAGC(5′biotin)	+

^1^The inhibitory sequence is underlined and bolded.

^2^The spaces are represent of deleted bases in the sequence.

^3^B = G/C/T, D = A/G/T, H = A/C/T, V = A/G/C, N = A/G/C/T.

^4^ +: strong inhibition. +/−: mild inhibition. −: no inhibition.

We proposed that Olig2.5 may have inhibitory effect to GXL DNA polymerase. To test this hypothesis, we used the primers Olig2F and OligTSSR to amplify the 2 kb fragment with an additional primer of Olig2.6 or Olig2.5. We found that adding of Olig2.6 doesn′’t inhibit the amplification of the 2 kb fragment, whereas adding of Olig2.5 blocked the amplification of the 2 kb fragment with GXL but not LA (Fig. 1C), supporting that Olig2.5 has an inhibitory effect to GXL DNAP. To further confirm the inhibitory effect of Olig2.5 to GXL DNA polymerase, we used a pair of primers Ubl35armF and Ubl35armR (Table 1) to amplify a 4.5 kb fragment unrelated to the 2 kb fragment amplified by Olig2F and OligTSSR. We found a similar inhibition of the PCR when Olig2.5 was added into the reaction (Fig. 1D).

To test if the inhibitory effect of Olig2.5 is dose dependent, we used Olig2F and OligTSSR and GXL DNAP to amplify the 2 kb fragment, and various concentrations of Olig2.5 was added into the PCR reactions. We found that the inhibitory effect is decreased when less amount of Olig2.5 is used (Fig. 1E). The inhibitory effect of Olig2.5 required a concentration higher than 0.05 μM (Fig. 1E). We thus raised a question that can we use the Olig2.5 at lower concentrations to decrease its inhibitory effect but keep its priming efficiency high enough? To answer this question, we used 0.4 μM of OligTSSR and various concentration of Olig2.5 to amplify the 2.5 kb fragment. The amplification of the 2.5 kb fragment is inefficient when Olig2.5 was used at the concentration of 0.2 μM. When the concentration of Olig2.5 was reduced to 0.1 μM, the 2.5 kb target was visible, and the amplification efficiency was further increased when the concentration of Olig2.5 was reduced to 0.05 μM (Fig. 1F). The dose-dependent inhibition of Olig2.5 to GXL was further confirmed by real-time PCR (Fig. 1G), which showed that Olig2.5 significantly inhibit PCR at concentrations higher than 0.05 μM.

### Primer Olig2.5 inhibits family B DNA polymerases

**Figure 2:**
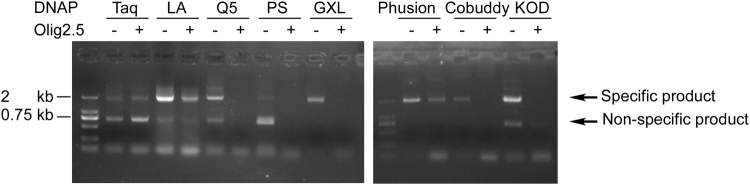
Olig2.5 inhibited archaeal family B DNA polymerases. An *E. coli* DH5α colony harbouring the 2.6 kb upstream sequence of *Olig2* in pGL3-Basic vector was cultured and 1 μl of the culture was directly used as template for PCR. Olig2F and OligTSSR were used as primers to amplify the 2 kb fragment. Olig2.5 was added to see its inhibition to various DNAP. DNAP: DNA polymerase. LA: LA Taq DNAP. Q5: Q5 High-Fidelity DNAP. PS: PrimeSTAR HS DNAP. GXL: PrimeSTAR GXL DNAP. Phusion: Phusion High-Fidelity DNAP. Cobuddy: Cobuddy Super Fidelity DNAP. KOD: KOD DNAP.

Taq and GXL belong to family A and family B DNA polymerases, respectively. LA is a Taq based enzyme mixture supplemented with a low level of a family B DNA polymerase. We asked if Olig2.5 could inhibit other family B DNAPs. Using bacterial culture harbouring the cloned 2.6 kb Olig2 promoter fragment as template DNA, Taq and LA can amplify the specific product and a non-specific product. The additional primer Olig2.5 doesn’t inhibit the amplification of the specific and non-specific products by Taq and LA (Fig. 2). Without Olig2.5, the specific product was successfully amplified by Q5, PS, GXL, Phusion, Cobuddy and KOD. Different from Taq and LA, when Olig2.5 was added to the PCR reaction, the amplification of the specific and non-specific products by Q5, PS, GXL, Phusion, Cobuddy and KOD were inhibited (Fig. 2). The results showed that the Olig2.5 is inhibitory to at least four family B DNA polymerases, Q5, PS (GXL is derived from PS by proprietary point mutation and PCNA enhancement), Cobuddy and KOD, suggesting that other family B DNA polymerases may be inhibited as well.

### TT(N)mGCCTC sequences caused inhibitory effect

**Figure 3:**
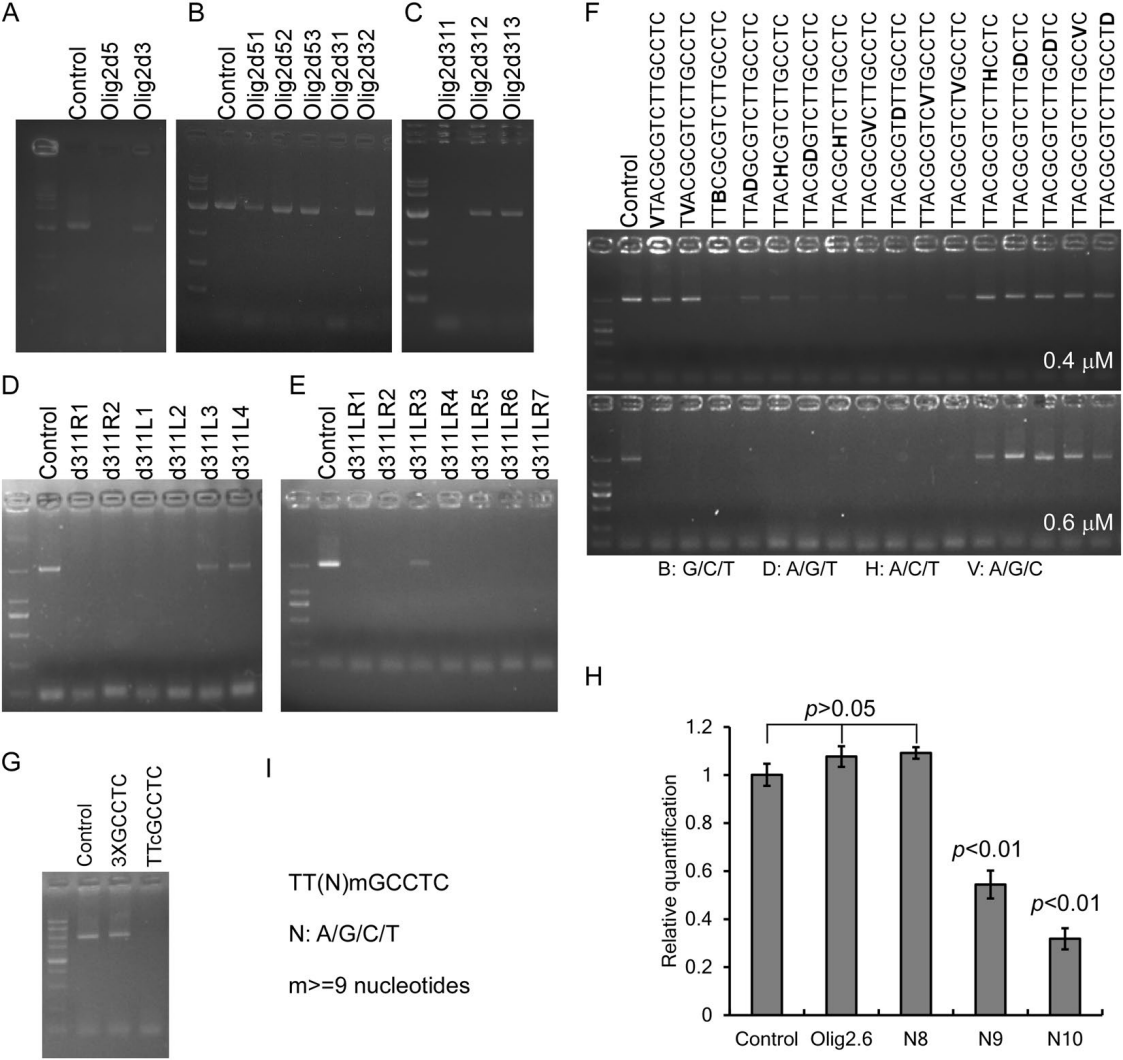
TT(N)mGCCTC sequence caused PCR inhibitory effect. (**A–E**) Testing PCR inhibitory effect of oligonucleotides derived from Olig2.5 by various deletion. The shortest inhibitory sequence is TTACGCGTCTTGCCTC. (**F**) Single nucleotide replacement revealed that oligonucleotides with mutations at the 5′ terminal TT and the 3′ terminal GCCTC have reduced PCR inhibitory effect. When used at a higher concentration of 0.6 μM, no apparent PCR inhibition was observed using oligonucleotides with mutations at the GCCTC. (**G**) The spaced TT and GCCTC are both required for PCR inhibition. 3XGCCTC and was tested to see their inhibitory effect. Triplicates of GCCTC failed to inhibit PCR. However, TTcGCCTC, an oligonucleotide with sequence between TT and GCCTC complement to TTACGCGTCTTGCCTC, inhibited PCR. (**G**) Real-time PCR showed that N9 and N10 significantly inhibited PCR efficiency with that N10 has a higher inhibition effect. (**H**) The minimal inhibitory sequence was concluded to be TT(N)mGCCTC.

To narrow down the sequence that causing the inhibitory effect to the family B DNAPs, we tested two oligonucleotides truncated from Olig2.5 to see if they can inhibit PCR (please see Table 1 for sequence information and PCR inhibitory effect of the tested oligonucleotides). The result showed that deletion of 8 nucleotides from the 5′ end of Olig2.5 (Olig2d5) still inhibits PCR, whereas deletion of 8 nucleotides from the 3′ end of Olig2.5 (Olig2d3) eliminated its inhibition (Fig. 3A). This means that a few nucleotides that is essential for PCR inhibition of Olig2.5 was deleted in Olig2d3. We then test several oligonucleotides derived from Olig2.5 with various deletion at the ends. We found that Olig2d51, deletion of 5 nucleotides from Olig2d5, didn’t inhibit PCR (Fig. 3B), suggesting inhibition sequence is initiated in the 5 nucleotides. Olig2d31 has 3 more nucleotides than Olig2d32, the different effect between Olig2d31 and Olig2d32 in PCR indicated that inhibition sequence is ended in the 3 nucleotides (Fig. 3B). To see if the inhibition sequence is a continuous one or not, we used 3 oligonucleotides derived from Olig2d31 by various deletion at the middle region. Although Olig2d312 and Olig2d313 didn’t showed PCR inhibition effect, Olig2d311 retains PCR inhibition effect (Fig. 3C), indicating that the inhibition sequence is a discontinuous one.

Based on Olig2d31, Olig2d32 and Olig2d311, we further test d311R1 and d311R2 to determine the 3′ end nucleotide required for PCR inhibition. Similarly, Olig2d5, Olig2d51 and Olig2d311, 4 oligonucleotides were used to determine the 5′ end nucleotide required for PCR inhibition. The results showed that d311R2 and d311L2 can inhibit the PCR (Fig. 3D). Together with the inability of Olig2d32 and d311L3 to inhibit PCR (Fig. 3B and D), the inhibitory sequence was shortened as d311LR1 (Table 1). Considering the differences of PCR inhibition between Olig2d311 and Olig2d312, we used d311LR1 and its derivatives to test their PCR inhibitory effect. All the derivatives by deletion of 1–4 nucleotides from d311LR1 could inhibit PCR (Fig. 3E). We further used a series of oligonucleotides with single nucleotide mutation to determine the minimum inhibitory sequence. When added at a final concentration of 0.4 μM, sequences with mutation of TT at the 5′ end and GCCTC at the 3′ end into other nucleotides did not cause PCR inhibition (Fig. 3F top). When used at 0.6 μM, only mutations at GCCTC didn’t cause PCR inhibition (Fig. 3F bottom). Finally, we found that triplicate repeats of GCCTC failed to inhibit PCR. However, TTcGCCTC, a complement sequence of d311LR1 between TT and GCCTC, still inhibited PCR (Fig. 3G). This indicated that both TT and GCCTC are required for PCR inhibition. We then used real-time PCR to determine the number of nucleotides required between TT and GCCTC to inhibit DNAP. We found that N10 inhibit PCR more severely than N9, and there is no inhibitory effect of oligonucleotide N8 (Fig. 3H). Taken together, these results suggested that TT(N)mGCCTC (Fig. 3I), where (N)m means any sequence equal or longer than 9 nucleotides, can inhibit PCR using archaeal family B DNAPs. It is interesting that the frequency of TT(N)_9–20_GCCTC in archaeal is higher than the estimated GCCTC frequency, whereas the frequency of TT(N)_9-20_GCCTC in bacterial is lower than the estimated GCCTC frequency (Table 2).

**Table 2:** Frequency of TT(N)_9-20_GCCTC.

	Bacterial				Archaeal			
	Escherichia coli		Thermus aquaticus		Thermococcus kodakarensis		Pyrococcus furiosus	
Genbank number	NC_000913.3		NZ_LHCI01000106.1		NC_006624.1		NC_003413.1	
Genome size	4641652 bp		2072904 bp		2088737 bp		1908256 bp	
%GC	50.79		67.34		52.00		40.77	
	**plus**	**minus**	**plus**	**minus**	**plus**	**minus**	**plus**	**minus**
TTN_9_GCCTC	171	165	177	178	180	207	142	143
TTN_10_GCCTC	182	198	335	359	244	262	114	164
TTN_11_GCCTC	170	144	272	276	187	204	136	137
TTN_12_GCCTC	161	154	187	178	169	169	183	178
TTN_13_GCCTC	190	160	413	417	257	247	184	172
TTN_14_GCCTC	173	173	276	325	234	193	164	181
TTN_15_GCCTC	139	171	194	173	226	179	169	192
TTN_16_GCCTC	187	198	395	399	301	297	184	191
TTN_17_GCCTC	174	177	328	299	248	242	188	194
TTN_18_GCCTC	149	160	185	190	182	207	210	178
TTN_19_GCCTC	188	186	377	414	238	288	159	155
TTN_20_GCCTC	182	171	301	305	223	199	164	156
TTN_9-20_GCCTC	2066	2057	3440	3513	2689	2694	1997	2041
GCCTC	2325	2374	9281	9568	3404	3442	1625	1596
^1^Frequency of TTN_9-20_GCCTC	2247	2257	603	590	777	775	956	935
^1^Frequency of GCCTC	1996	1955	223	217	614	607	1174	1196
^2^Estimated GCCTC frequency	977	977	477	477	912	912	1955	1955

^1^Frequency = Genome size/number of TTN_9-20_GCCTC or GCCTC.

^2^Estimated GCCTC frequency = 1/2 × (%GC)^4^ (1-%GC)

### TT(N)mGCCTC-containing sequence binds to family B DNA polymerase but not Taq DNA polymerase

**Figure 4:**
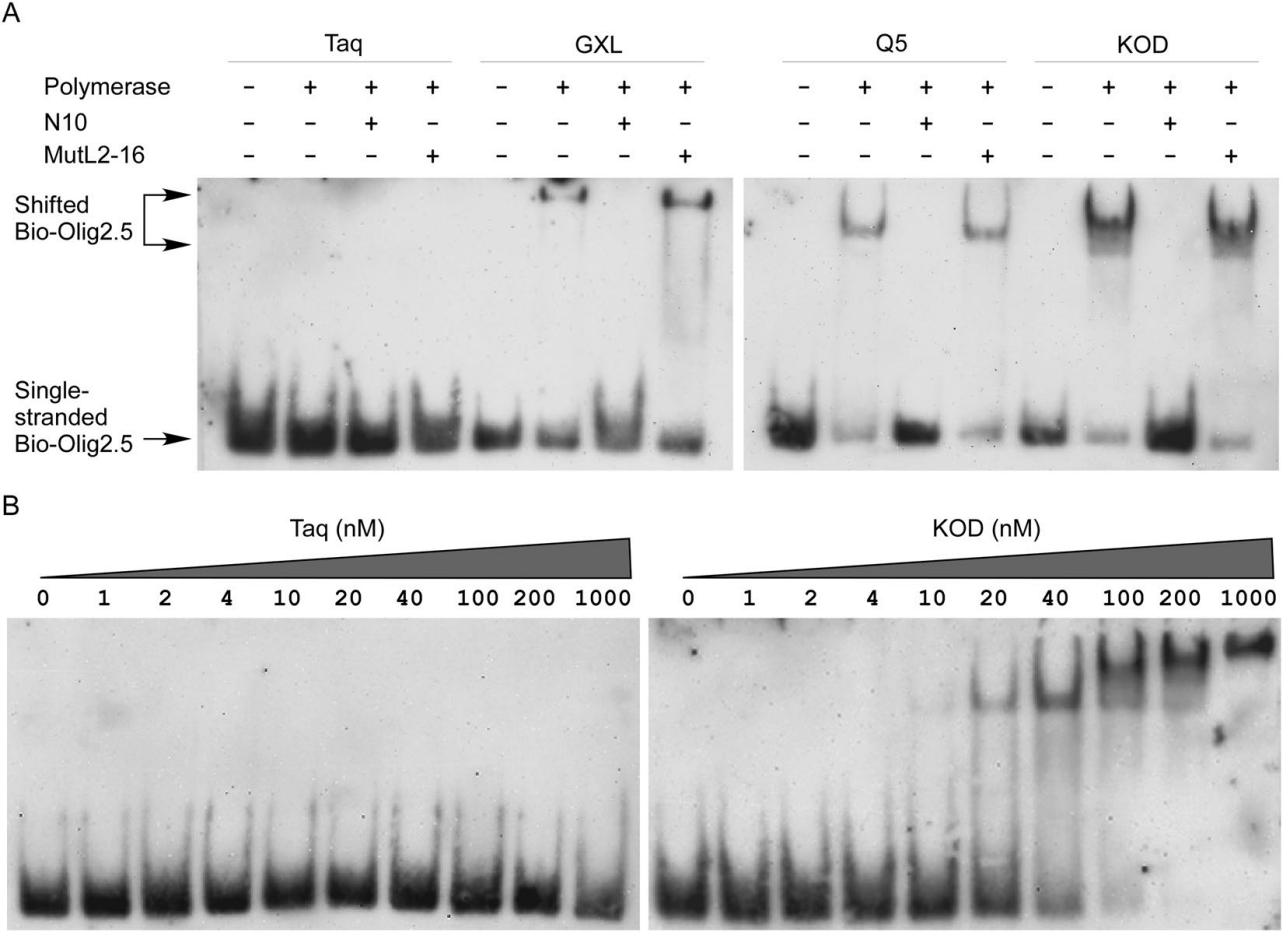
Proofreading DNA polymerases but not Taq DNA polymerase bind to Olig2.5. (**A**) Biotin-labeled oligonucleotide Bio-Olig2.5 was subjected to electrophoresis mobility shift assay. All DNAPs except Taq caused band retardation. The retarded bands caused by GXL, Q5 and KOD were disappeared by adding 1000 × excess amount of specific competitor N10 but not non-specific competitor MutL2-16. (**B**) Taq didn’t cause any band shift even used at 1000 nM. A shifted band appeared when KOD was used at 10 nM approximately half of the Bio-Olig2.5 was shifted when KOD was at 40 nM. Full-length membrane exoposure image is presented in Supplementary Figure 2.

To investigate if TT(N)mGCCTC-containing sequences can bind to and thus inhibit the family B DNA polymerases, we performed electrophoresis mobility shift assay (EMSA). Taq didn’t cause any band shift of the Bio-Olig2.5 bands (Fig. 4A), indicating that without Mg^2+^ the affinity between Taq and single-stranded DNA is too low to be detected. However, a shifted band of Bio-Olig2.5 was detected when there is a family B DNA polymerase such as GXL, Q5 and KOD (Fig. 4A). The shifted bands disappeared when excess amount of N10 was added as unlabeled specific competitor (Fig. 4A). However, the shifted bands persisted even there was 1000 excess amount of MutL2-16 as non-specific competitor, indicating that proofreading DNA polymerases specifically bind to TT(N)mGCCTC-containing sequences. We finally used purified Taq and KOD to measure the equilibrium dissociation constant (K_D_) between DNAPs and Olig2.5. Our results showed that approximately 50% of the Olig2.5 was in a complex with KOD when the latter is at 40 nM (Fig. 4B), suggesting a K_D_ value of about 40 nM. In contrast, there’s no detectable interaction between Olig2.5 and Taq even Taq was used at a concentration as high as 1000 nM (Fig. 4B).

## Discussion

We here showed that TT(N)mGCCTC-containing oligonucleotides can specifically inhibit PCR using proofreading family B DNAPs. A few DNA aptamers that can inhibit DNAPs have been identified^17–19^. These aptamers bind to and inhibit DNAPs at relatively low temperature through their secondary structures or G-quadruplex structures. As these structures are normally disrupted during PCR, these aptamers can be used to increase PCR specificity. As TT(N)mGCCTC cannot form a stable secondary structure, the inhibition effect of TT(N)mGCCTC to proofreading DNAPs relies on its primary structure. To the best of our knowledge, this is the first report describing of archaeal family B DNAPs inhibited by the single-stranded form of oligonucleotides.

PCR may fail if a TT(N)mGCCTC-containing primer was used together with a proofreading DNA polymerase, as the primers are normally used at a final concentration higher than 0.1 μM. Researchers tend to add more primers to increase the amplification efficiency by enhancing the template-primer binding, which will further decrease the efficiency of PCR if proofreading DNA polymerases and TT(N)mGCCTC-containing primers are used. In such cases, use the inhibitory primers at lower concentrations such as 0.1 μM and 0.05 μM will greatly improve the PCR yield, as the inhibition effect of TT(N)mGCCTC-containing primers are not significant when they are used below 0.1 μM (Fig. 1D&E). The user manual of KOD-Plus-Neo (http://www.toyobo-global.com/seihin/xr/lifescience/support/manual/KOD-401.pdf) showed that higher concentrations of primers may inhibit PCR amplification of long targets. The long target bands was already weakened before the concentration of primers are high enough to introduce non-specific amplification of shorter fragments, indicating that the inhibition is not due to the non-specific amplifican. Possibly there are sequences other than the TT(N)mGCCTC described here, and the previously described GGGGG and GGGGHGG that can inhibit proofreading DNA polymerases^16^. The use of lower concentration of primers could be a general rule if a proofreading DNA polymerase failed to amplify a target.

It is well known that the sequence 5′-GAGGC-C-GAGGC-C-GCCTC-G-GCCTC-3′, consisting of four GAGGC/GCCTC repeats, are essential for the large-T-antigen binding and DNA replication of simian virus 40 (SV40)^20,21^. Here, the GAGGC sequence is reverse complement to GCCTC. The four GAGGC/GCCTC repeats are also present in the origin of DNA replication of polyoma virus JC and polyoma virus BK^22^. The large-T-antigen can bind to GAGGC/GCCTC repeats and unwind the duplex DNA^20,21^. Protein-protein interaction between the large-T-antigen and DNA polymerase α, a eukaryotic family B DNAP, directly stimulate the replication of the SV40 genome^23^. We don’t know if DNA polymerase α utilizes a distantly located TT from GCCTC for its DNA binding and replication. Although the frequency of TT(N)mGCCTC is higher than expected in archaeal genome (Table 2), if there is any biological function of the sequence TT(N)mGCCTC, which inhibits archaeal family B DNAPs *in vitro*, remains elusive *in vivo*.

## Methods

### Thermostable DNA polymerases

The following thermostable DNA polymerases were used in this study. Taq DNA polymerase (Taq) was purchased from Sangon Biotech (Shanghai, China). LA Taq™ Version 2.0 (LA), PrimeSTAR (PS) and PrimeSTAR^®^ GXL DNA Polymerase (GXL) were from TaKaRa Bio (Dalian, China). Phusion^®^ High-Fidelity DNA Polymerase (Phusion) and Q5^®^ High-Fidelity DNA Polymerase (Q5) were from New England Biolabs. Cobuddy Super Fidelity DNA Polymerase (Cobuddy) was purchased from CWBiotech (Beijing, China). KOD DNA polymerase (KOD) was purchased from TOYOBO Biotech (Shanghai, China). Taq belongs to family A DNAP, whereas PS and KOD belong to family B DNAP. LA is composed of KlenTaq (an N-terminal deleted version of Taq) and a small portion of a family B DNAP. GXL is modified from PS with proprietary point mutation and enhanced probably by proliferating cell nuclear antigen. Q5 and Cobuddy are Sso7d-fused family B DNAPs.

Plasmids pTrc-Taq and pET22b-KOD were purchased from Miaoling Bioscience & Technology (Wuhan, China). His tag was cloned into pTrc-Taq to generate pHis-Taq plasmid using T4 DNA polymerase^24^. Briefly, 50 ng of NcoI digested pTrc-Taq plasmid treated with 1 unit of T4 DNA polymerase at 20 °C for 3 min, followed by 75 °C for 5 min to inactivate T4 DNA polymerase, then oligonucleotides HisInTaqF and HisInTaqR (Table 1) were added into the reaction during the 75 °C incubation, the reaction was incubated at 50 °C for 10 min before transformation into competent cells. pHis-Taq and pET22b-KOD were transformed into Rosetta (DE3) for expression, and His-tagged Taq and KOD were purified by Ni-NTA-Sefinose Column (Sangon Biotech). Purified Taq and KOD were used for measuring DNA binding constant.

### PCR conditions

PCR primers are listed in Table 1. PCR programs for Q5, Cobuddy, PS, GXL and KOD is: initial denaturation at 98 °C for 30 s; followed by 25–35 cycles of denaturation at 98 °C for 10 s, and annealing and extension at 66 °C for 30 s/kilobase (kb); and a final extension at 66 °C for 5 min. The same program except that annealing and extension for 50 s/kb was used for LA. For Taq, the initial denaturation was at 94 °C for 3 min, and the cycling was 94 °C for 30 s, and 66 °C for 50 s/kb. All PCRs were carried out in a total volume of 25 μl. The amount of enzymes used in PCR were 1.25 U, 1 U, 0.625 U, 0.625 U, 1 U, 1 U and 70 ng for Taq, Q5, PS, GXL, Phusion, Cobuddy and KOD respectively. Taq reaction buffer contains 10 mM Tris, pH8.3, 50 mM KCl, 1.5 mM MgCl_2_ and 0.1% Triton-X100. KOD reaction buffer contains 50 mM Tris, pH8.8, 10 mM KCl, 15 mM (NH_4_)_2_SO_4_, 2 mM MgCl_2_ and 0.1% Triton-X100. Buffer information for the other enzymes are unavailable. All PCR used a final concentration of 0.2 mM of dNTPs except LA, which is undisclosed. A homemade 10 × GC enhancer consisting of 2.5 M of betaine, 1 M of trehalose and 12.5% (v/v) of DMSO was used to enhance PCR performance. Primers were used at a final concentration of 0.4 μM unless specified. Each 50 ng of mouse genomic DNA or 1 μl of cultured bacterial medium was used as template. Each 5 μl of PCR product was used for electrophoresis. Each 5 μl of PCR product was used for electrophoresis. For real-time PCR, Olig2qF and Olig2qR were used as primers, and a final concentration of 0.25 × SYBR Green I (Thermo Fisher Scientific) was included in the PCR reaction. One-way ANOVA with post-hoc Tukey HSD was used for statistical analysis based on 3 independent experiments.

### Electrophoresis mobility shift assay (EMSA)

Each 50 fmol of 5′-Biotin labeled oligonucleotide Bio-Olig2.5 (please see Table 1 for sequences of Bio-Olig2.5) was mixed with 1 U of Taq, Q5, GXL or 150 ng of KOD in 1X Binding Buffer (10 mM Tris-HCl pH 7.9, 50 mM KCl, 1 mM EDTA, 5% glycerol, 1 mM DTT, and 25 μg/ml BSA), and 50 pmol of N10 and MutL2-16 was used as specific competitor and non-specific competitor, respectively. The binding reaction was performed without MgCl_2_ and Poly (dI-dC). After 20 min incubation at room temperature, the samples were mixed with 5X Loading Buffer (0.25X TBE buffer, 30% glycerol, and 0.2% bromophenol blue) and loaded onto 6% polyacrylamide gels in 0.5X TBE. To measure the equilibrium dissociation constant (KD) of DNAP and Olig2.5 interaction, 2 nM Bio-Olig2.5 was mixed with various concentration of purified KOD or Taq in a total volume of 20 μl^25^. Oligonucleotides were electrophoresed and transferred onto Immobilon-Ny^+^ membranes (Millipore). Chemiluminescent detection was performed according to the user manual of Chemiluminescent EMSA Kit (Beyotime Biotechnology, Nantong, China).

## Electronic supplementary material

Supplementary Information
