# Supplementary material for: TT(N)mGCCTC inhibits archaeal family B DNA polymerases
Source: Sci Rep. 2018 Jan 31;8:1990. doi: 10.1038/s41598-018-20127-4 (PMC5792436; doi:10.1038/s41598-018-20127-4)
Supplement: Supplementary file 1 — Supplementary Information [file 41598_2018_20127_MOESM1_ESM.doc]

**TT(N)mGCCTC inhibits archaeal family B DNA polymerases**

Shuhui Sun^1,2^, Wei Guo^1,2^, Jin-Shu Yang^1^, Mengsheng Qiu^2,3^, Xiao-Jing Zhu^2^* and Zhong-Min Dai^2^*

^1^ College of Life Sciences, Zhejiang University, 866 Yuhangtang Road, Hangzhou, Zhejiang, 310058, PR China

^2^ Institute of Life Sciences, Key Laboratory of Organ Development and Regeneration of Zhejiang Province, College of Life Sciences, Hangzhou Normal University, Hangzhou, Zhejiang, 310036, PR China

^3^ Department of Anatomical Sciences and Neurobiology, University of Louisville, Louisville, KY40292, USA

* To whom correspondence should be addressed. Tel: 86-571-28866385; Email: zhongmindai@hznu.edu.cn; xiao_jingzhu@hotmail.com


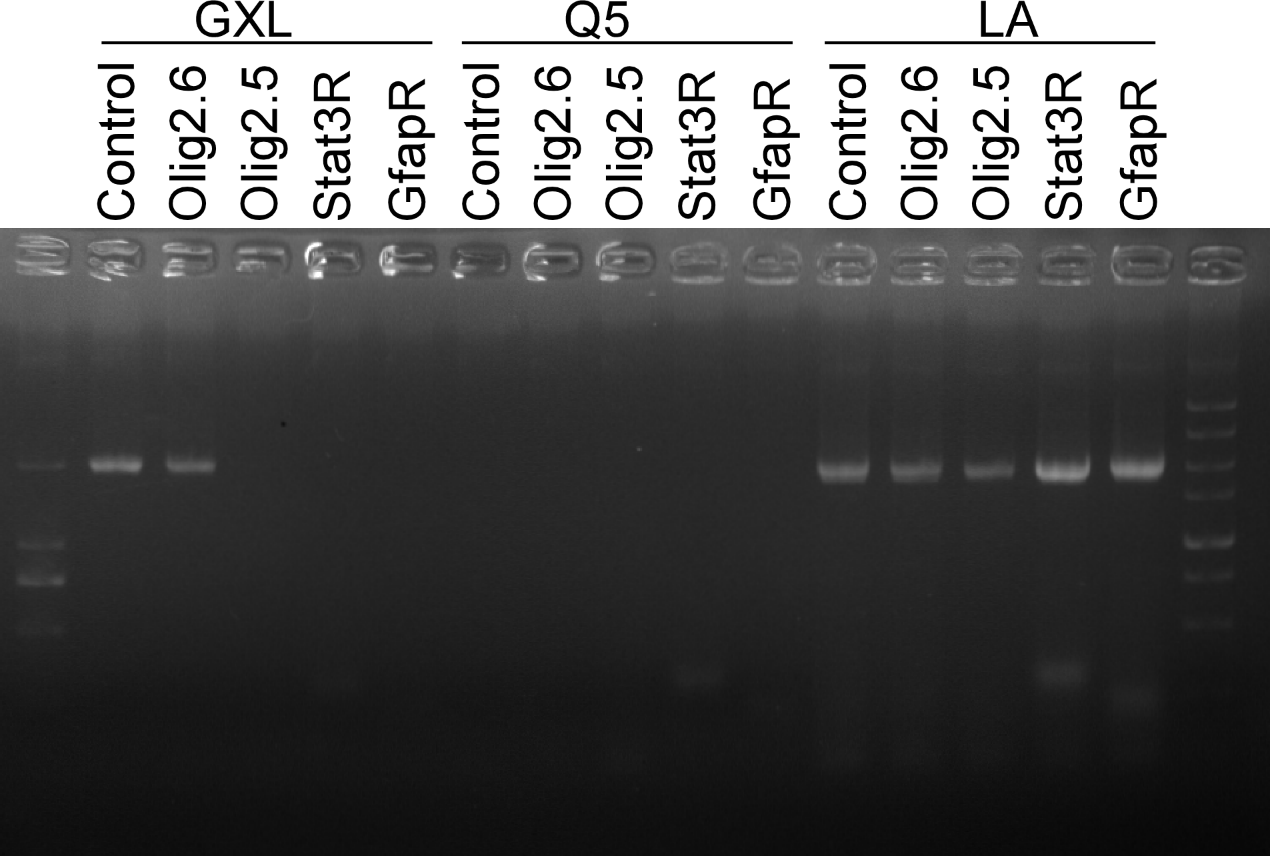


Supplementary Figure 1. Full-length gel of Fig. 1C


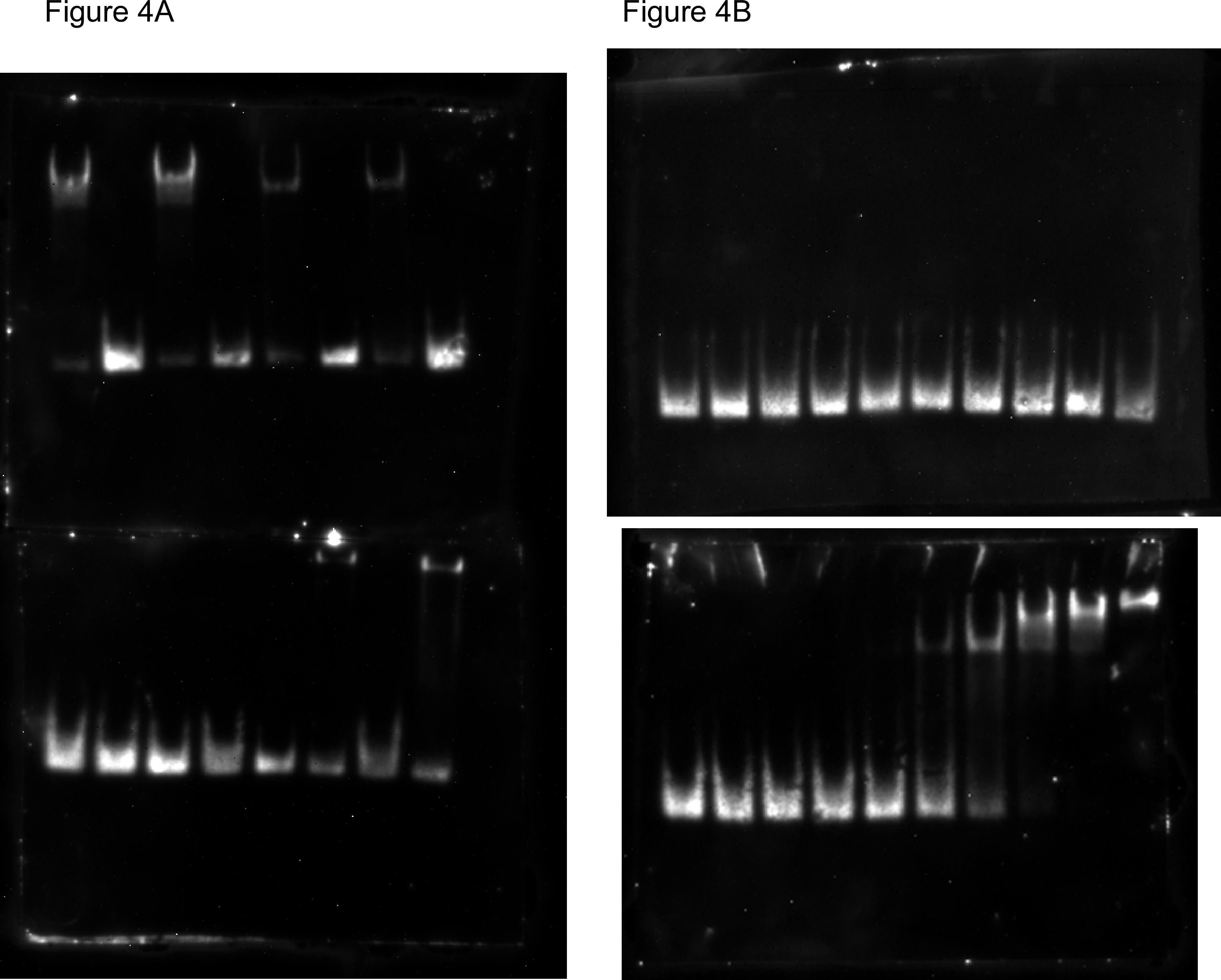
Supplementary Figure 2. Full-length membrane of Fig. 4.
